# Nicotine Pouch: Awareness, Beliefs, Use, and Susceptibility among Current Tobacco Users in the United States, 2021

**DOI:** 10.3390/ijerph20032050

**Published:** 2023-01-22

**Authors:** Lindsey S. Sparrock, Lilianna Phan, Julia Chen-Sankey, Kiana Hacker, Aniruddh Ajith, Bambi Jewett, Kelvin Choi

**Affiliations:** 1 Department of Neuroscience, American University, Washington, DC 20016, USA; 2 Division of Intramural Research, National Institute on Minority Health and Health Disparities, Bethesda, MD 20892, USA; 3 Center for Tobacco Studies, Rutgers Biomedical and Health Sciences, New Brunswick, NJ 08901, USA; 4 School of Public Health, Rutgers Biomedical and Health Sciences, Piscataway, NJ 08854, USA; 5 University of Pittsburgh School of Medicine, Pittsburgh, PA 15261, USA

**Keywords:** nicotine pouch, awareness, perceptions, tobacco use

## Abstract

Little is known about awareness, beliefs, and use of nicotine pouches (NPs). Data from 1583 U.S. adult (age ≥ 21 years) current tobacco users were collected in 2021. Respondents self-reported NP awareness, beliefs, use, and susceptibility as well as current tobacco product use and socio-demographics. We used weighted logistic and multinomial regression models to explore the associations between these variables. Overall, 46.6% of U.S. adult current tobacco users were aware of, 16.4% had ever used, and 3.0% currently used NPs. Younger individuals (vs. 61+ years) were more likely to have ever heard of NPs, while Black individuals (vs. White) were less likely to have ever heard of NPs. Individuals younger than 45 years (vs. 61+ years) and those using smokeless tobacco products (vs. non-users) were more likely to have ever used NPs. Additionally, younger than 45 years (vs. 61+ years) and current use of certain tobacco products (e.g., smokeless) were associated with current NP use. Holding favorable beliefs about NPs was associated with susceptibility to and more advanced NP use statuses (*p* < 0.05). Continuous surveillance of NP use and beliefs is important.

## 1. Introduction

Nicotine pouches (NPs) are an emerging class of noncombustible nicotine products. These thin, prefilled, microfiber pouches contain white powdered nicotine. Similar to Swedish-style snus, NPs are placed between the upper lip and gum, where the nicotine dissolves in the mouth without requiring spitting (e.g., [1,2]). In contrast with Swedish-style snus and other traditional smokeless tobacco products, NPs do not contain any tobacco leaf [3]. Rather, NPs typically contain nicotine salts, which deliver higher levels of nicotine than the free-base nicotine in most smokeless tobacco products [1], and vary widely in their nicotine content per pouch [4]. Other common ingredients in NPs include stabilizers (hydroxypropyl cellulose), fillers (microcrystalline cellulose, maltitol, and gum arabic), pH adjusters (sodium carbonate and sodium bicarbonate), sweeteners (acesulfame K). A variety of food-grade flavorants (e.g., fruit, mint, coffee) are also typically added to NPs [1,5] to make them more appealing. Currently, popular NP brands include Zyn (Swedish Match North American), On! (Altria), Velo (RJ Reynolds [RJR] Vapor Company/British American Tobacco [BAT]), Dryft (Kretek International), and Nordic Spirit (Japan Tobacco International) [6].

NPs have been promoted as cost effective (comparable to a pack of cigarettes [7]; convenient (as they can be used anywhere and do not require batteries or a device [8,9]; and relatively safe in comparison to other nicotine products (e.g., [5,10,11,12]). As of 2021, the global NP market was valued at USD 1.50 billion [13]. Based on the steady increase in sales, industry analysts predict that NPs will be valued at USD 22.98 billion by 2030 [14]. While NPs are currently available in many countries, the industry claimed that the largest NP markets are the US and Sweden [15], which may be due to the high prevalence of smokeless tobacco product use in these countries: 2.4% in the U.S. in 2019 [16], and 12.3% in Sweden in 2010 [17]. NPs entered the US market in 2016 and have since increased considerably in sales (from USD 709,635 in 2016 to USD 216,886,819 in 2020; [18].

Industry-funded research has claimed NPs as a “reduced-risk” product [19] and argued that these products have lower in vitro toxicity compared with conventional tobacco products, partially due to the absence of tobacco leaf and combustion [20]. However, these products have raised concerns among public health officials. These concerns include NP companies’ aggressive and targeted marketing strategies [1,5,21]. Some NP manufacturers may be practicing unethical marketing approaches and targeting individuals who do not use commercial tobacco and young people who are especially vulnerable to nicotine addiction [22,23]. This is particularly seen with advertisements depicting young adult models [24], as well as some brands advertising their products as “flavor ban approved” [25] or “tobacco-free” [1]. Current research also found that NPs contain carcinogens (e.g., tobacco-specific nitrosamines; [26], potentially high nicotine content [26], and/or similar concentrations and delivery speeds as other smokeless tobacco products [3], and side effects (e.g., nausea, hiccups, oral soreness, or irritation) [27]. As such, there remains uncertainty on the immediate and long-term NP-related health risks and public health impact.

In light of these circumstances, evaluations of awareness, use, and beliefs about NPs are important to monitoring the evolving NP and tobacco landscape. However, few existing studies have evaluated these measures. In the UK, Brose and colleagues (2021) examined the prevalence and correlates of NP use in a cohort of adult current or former smokers and/or electronic vaping product (EVP) users in 2019 [28]. They found that 15.9% of respondents were aware of NPs, 4.4% had ever used, and 2.7% were currently using NPs. A more recent study between November 2020 and October 2021 among adults (≥18 years) in Great Britain (England, Scotland, and Wales) found that 0.21% of respondents were currently using NPs [29]. Furthermore, a 2020 study of Dutch adolescents and adults (≥13 years) found that 6.9% were aware of NPs, 0.6% had ever used NPs, and 0.1% were currently using NPs [30]. In the US, only three studies examined the prevalence of awareness, ever, and current use of NPs. Felicione and colleagues (2022) surveyed a cohort of adult former and current cigarette smokers and EVP users in 2020 and found that 19.5% were aware of NPs, 3.0% ever used NPs, and 0.9% currently used NPs [31]. Another study of US adult smokers in early 2021 found a higher prevalence of awareness and ever use measures: 28.2% were aware of NPs and 5.6% ever used NPs (this study did not assess current NP use [32]). 

Understanding susceptibility to NPs is also important and must be understood in order to guide regulatory decision making and prevent increased initiation of these products. Susceptibility has been shown to significantly predict smoking initiation above and beyond other known risk factors (e.g., family members’ or peers’ smoking) or demographic factors (e.g., sex, race, SES) (e.g., [33,34,35]). Subsequent research has shown that there is a significant overlap in susceptibilities across various tobacco products [36]. Likewise, research examining the predictive validity of adapted susceptibility measures on alternative tobacco products (e.g., e-cigarettes, cigarettes, hookah, and cigars/cigarillo/little cigars) found that for each tobacco product, susceptibility predicted future initiation, suggesting that valid adapted susceptibility measures can be developed for different tobacco products [37]. While many studies have investigated the susceptibility to emerging nicotine and tobacco products, only two recent studies have specifically looked at NPs (e.g., [38,39]), although these were with adolescent and young adult samples, respectively. 

Studies are needed to track the prevalence of NP awareness, experimentation, and current use in the US given the rapid sales expansion. Examining the correlates of awareness and use is also necessary to shed light on what groups may be using these products. Moreover, to date, only one study has investigated beliefs about NPs and how these beliefs relate to NP susceptibility and use [39]. Understanding these beliefs is important for public health officials to know if there are any misbeliefs regarding the safety of these products and adequately address them. Furthermore, understanding how these beliefs relate to NP susceptibility is useful in informing efforts to prevent susceptible individuals from progressing to experimentation. Therefore, the aim of the present study was to investigate NP awareness, use, susceptibility, and beliefs among a 2021 nationally representative sample of US adult current commercial tobacco users to address these research gaps.

## 2. Methods

### 2.1. Study Population

We analyzed data from the COVID-19 and Commercial Tobacco Use Study (CaCTUS), an online cross-sectional survey of a nationally representative sample of U.S. adults who reported being recent former or current commercial tobacco users conducted in January–February 2021 [40]. Inclusion criteria were: (1) residing in the U.S.; (2) adults aged ≥ 21 years; and (3) currently used commercial tobacco or have used commercial tobacco during the 12 months prior to the study (including cigarettes, cigars, e-cigarettes, hookah, and other combustible tobacco products, and smokeless tobacco products). Survey respondents were recruited from the YouGov online survey panel. YouGov constructed a sampling frame to meet study inclusion criteria by utilizing a sample-matching approach based on data from nationally representative surveys. This approach allows for similar levels of representation as the random-digit-dialing sampling approach [41]. Subsequently, demographics and commercial tobacco use history distributions of the sampling frame were utilized to recruit a sample from YouGov online panelists with matching distributions. To ensure stable statistical estimates among Black and Asian/Asian American adults, these populations were oversampled. 

### 2.2. Study Procedures

YouGov online panelists were invited to participate via email. Interested individuals completed eligibility screening, and those who were eligible were then invited to complete the survey after providing informed consent. Respondents were asked about their NP awareness, use, beliefs, and susceptibility, in that order. Respondents were compensated in accordance with YouGov policy. Among the 2404 eligible panelists, 2123 completed the survey (completion rate = 88.3%). A final sample of 1700 respondents was obtained after excluding those who provided inconsistent responses or who were in groups that exceeded the study quotas. The analytic sample was restricted to 1583 respondents who reported currently using commercial tobacco at the time of survey. This analysis did not require a review by the National Institutes of Health Institute Review Board per 45 CFR 46 as this analysis did not involve identified data and thus is considered “not human subjects research”.

### 2.3. Measures

#### 2.3.1. NP Awareness, Use, and Susceptibility

Respondents were presented with an image of some popular brands of nicotine pouches (e.g., Dryft, On, Velo, and Zyn) and asked the following questions: “Have you ever seen or heard of nicotine pouches before this study?” Those who answered “yes” were classified as being aware of NPs, and those who answered “no” or “not sure” were classified as being unaware of NPs. Respondents who were aware of NPs were then asked, “Which of the following best describes your experience with nicotine pouches?” (Response options: never used it before, used it before but not currently, currently using it some days or every day.). Additionally, all respondents were asked NP susceptibility measures: “Are you curious about nicotine pouches?”, “Do you think you will try nicotine pouches soon?”, “Do you think you will try nicotine pouches in the next year?”, and “If one of your best friends were to offer you some nicotine pouches, would you use them?” (Response options: definitely not, probably not, probably yes, definitely yes). Respondents who answered “definitely not” for all four questions were classified as non-susceptible to NP use. Otherwise, respondents were classified as susceptible to NP use. Based on their responses to the NP use and susceptibility measures, respondents were further categorized into four groups: non-susceptible never NP users (non-susceptible and never used NPs), susceptible never NP users (susceptible but never used NPs), ever-non-current NP users (used NP before but not at the time of survey), and current NP users (used NPs at least some days at the time of survey). 

#### 2.3.2. NP Beliefs

Respondents were randomized to view one of three images: two showed advertisements of ZYN, and one showed a white plate. They were then asked to rate their agreement with the following statements: “Nicotine pouches are less harmful than other smokeless tobacco e.g., chewing or dipping tobacco”, “Nicotine pouches are less addictive than other smokeless tobacco e.g., chewing or dipping tobacco”, “Using nicotine pouches occasionally does not cause any harm to the users”, “Using nicotine pouches occasionally does not cause users to be addicted to nicotine”, and “Nicotine pouches are for someone like me”. To avoid ordering effects, these statements were presented in random order. Responses were categorized as “agree” (including “strongly agree” and “somewhat agree”), “disagree” (including “strongly disagree” and “somewhat disagree”), and “don’t know”.

#### 2.3.3. Socio-Demographics and Commercial Tobacco Use Statuses

Socio-demographic information, including age, sex, race/ethnicity, educational attainment, annual household income, and urbanicity, was collected since they have been previously shown to be associated with commercial tobacco use (see Table 1 for categories of these variables). Respondents reported their age (in years), and these responses were categorized into age groups. They also chose from a list the best race/ethnicity representing them. Due to sample sizes in some response categories, those reported as Middle Eastern or North African, Pacific Islander, American Indian or Alaskan Native, and multiracial/multiethnic were grouped as other. Educational attainment was based on self-report highest grade or year of school completed. Respondents also reported their annual household income from all members and all sources in the household. Urbanicity was a derived variable provided by YouGov. Current use (i.e., currently using the product some days or every day) of the following tobacco products at the time of survey was also assessed: cigarette, electronic vaping products, cigars (including premium cigar, cigarillos, and little filtered cigars), hookah, other combustibles, and smokeless tobacco. 

### 2.4. Statistical Analyses

Post-stratification weights were applied in all analyses to achieve a national representation of US adults who currently and formerly used tobacco. Sociodemographics and tobacco product use statuses of the sample were summarized in weighted percentages. Prevalence estimates of awareness of NPs and NP use, as well as NP-related beliefs overall and by sociodemographic and tobacco product use status, were calculated. Three sets of weighted logistic regression models were fitted. First, we modeled NP awareness as the dependent variable (aware vs. unaware), and sociodemographics as the independent variable to examine the association of sociodemographics with NP awareness. Second, we modeled NP awareness as the dependent variable, tobacco product use statuses as independent variables, and sociodemographics as covariates, to examine the association of tobacco product use statuses with NP awareness. We used this approach so that we did not dilute the association between sociodemographics and NP awareness and use, since sociodemographics can be causally related to NP awareness and use through other tobacco product use. The same two-step logistic regression modeling approach was used to model NP ever use (yes vs. no) and NP current use (yes vs. no) as dependent variables, and sociodemographic and tobacco product use statuses as independent variables. Lastly, weighted multinominal regression was conducted to examine the associations between NP-related beliefs and NP susceptibility and use statuses. Each belief was modeled separately, using “disagree” as the reference, adjusting for socio-demographics and tobacco product use statuses. For example, to examine the associations between believing NP is less harmful than smokeless tobacco and NP susceptibility and use statuses, a multinomial regression model was fitted, using a 4-level variable (non-susceptible never vs. susceptible never, ever not current, current) as the dependent variables, the 3-level belief variable (agree, don’t know vs. disagree) as the independent variable, and socio-demographics and other tobacco products use statuses as covariates. Since beliefs did not differ significantly by the randomized images respondent saw and NP use statuses, we did not include the image seen as a covariate in the model. Analyses were conducted in SAS^®^ Enterprise version 9.4 (SAS Institute, Inc., Carey, North Carolina, USA). 

## 3. Results 

### 3.1. Prevalence and Correlates of NP Awareness and Use

Table 1 summarizes the weighted characteristics of the study population. It also shows the prevalence of awareness, ever use, and current use of NP by socio-demographics and tobacco product use statuses, as well as the adjusted odds ratios (AORs) between these variables and NP awareness and use. Overall, 46.6% (n = 710) were aware of NPs, 16.4% (n = 278) ever used NPs, and 3.0% (n = 63) currently used NPs. Results from the multivariable logistic regression models revealed that individuals younger than 61 years of age (vs. 61+ years) were more likely to be aware of NPs, and Black individuals (vs. White) were less likely to be aware of NPs (*p* < 0.05). Additionally, individuals between 18–45 years (vs. 61+ years) and smokeless tobacco users (vs. non-users) were more likely to have ever used NPs (*p* < 0.05). Individuals between 18–45 years (vs. 61+ years) who reported current use of electronic vaping products (vs. non-use), current use of cigarettes (vs. non-use), and current use of smokeless tobacco products (vs. non-use) were more likely to be currently using NPs (*p* < 0.05).

### 3.2. NP Beliefs, NP Susceptibility and Use Status

Table 2 shows the prevalence of NP beliefs and their associations with NP use statuses. Overall, 23.2% believed that NP is less harmful than smokeless tobacco, 16.1% believed that NP is less addictive than smokeless tobacco, 18.9% believed that using NP occasionally is not harmful, 14.6% believed that using NP occasionally is not addictive, 33.2% believed that NP is socially acceptable, and 19.1% believed that NP is for someone like themselves. Results from the multivariable multinomial regression models showed that holding favorable beliefs about NPs was associated with more advanced NP use statuses when disagreeing with a belief and being a non-susceptible NP user was the reference. For example, those who agree that NP is less harmful than smokeless tobacco had higher odds of being susceptible never NP users (AOR = 4.88, 95% CI = 2.44, 9.77), ever-not-current NP users (AOR = 2.91, 95% CI = 1.33, 6.34), and current NP users (AOR = 10.61, 95% CI = 3.44, 32.78). Additionally, those who were unsure about the harm and addictiveness of NPs had lower odds than ever-but-not-current NP users and current NP users. For example, those who reported “don’t know” about using NP occasionally is not harmful had lower odds of being ever-but-not-current NP users (AOR = 0.37, 95% CI = 0.19, 0.72) and current NP users (AOR = 0.05, 95% CI = 0.01, 0.30).

## 4. Discussion

The present study provided estimates on the prevalence of NP awareness, ever use, and current use among a nationally representative sample of US adult current commercial tobacco users. Overall, 46.6% of these adults reported awareness of NPs, 16.4% reported ever use, and 3.0% reported current use in 2021. We are aware of two studies to date that have examined awareness and/or use of NPs among US adult current and former tobacco users [31,32]. Felicione and colleagues (2022) assessed awareness and use prevalence among US current and former tobacco users in 2020, finding that 19.5% of respondents had ever heard of, 3% had ever used, and 0.9% were currently using NPs [31]. Similarly, Hrywna and colleagues (2022) examined NP awareness, interest, and ever use among a nationally representative sample of US adult current smokers in 2021 [32]. They found that 29.2% of respondents had ever heard of, 16.8% had interest in using in the next 6 months, and 5.6% had ever used. Our findings suggest that awareness of, experimentation with, and current use of NPs drastically increased between 2020 and 2021. This may be attributed to the notable increase in NP marketing by manufacturers, suggesting that the strategies implemented by the tobacco industry are working. These strategies include advertising on radio and television, as well as mobile and online displays [42]. It is noteworthy that while the NPs are under the jurisdiction of the US Food and Drug Administration (FDA), they were not included in the Comprehensive Smokeless Tobacco Health Education Act of 1986, and are therefore still allowed to advertise on radio, television, or other media, unlike conventional smokeless tobacco products [43]. Implementing federal policies to regulate NP advertising will close this loophole that is being exploited by NP companies, especially when there is no evidence that NPs are replacing combustible tobacco products or conventional smokeless tobacco.

Our study is the first to examine beliefs on NPs’ absolute harm and addictiveness, their relative harm and addictiveness to smokeless tobacco, and their social acceptability; further, this is the first study to investigate the relationships between these beliefs and NP susceptibility and use statuses. Vogel and colleagues (2022) surveyed a cohort of young adults (19–23 years) from Southern California to examine the perceived relative harm between NPs, cigarettes, and e-cigarettes [44]. They found that 19.7% of their sample believed NPs to be less harmful than cigarettes, and 13.6% believed NPs to be less harmful than e-cigarettes. In our study, we found that between 14.6% (using NP occasionally is not addictive) and 33.2% (NP is socially acceptable) of US adult current commercial tobacco users held favorable beliefs about NPs. Holding these beliefs was associated with susceptibility to and more advanced/higher levels of NP use. These findings are supported by a previous study showing that favorable NP beliefs were related to susceptibility to NP use and NP awareness [39]. While our findings suggest that holding specific favorable beliefs is associated with NP susceptibility and use, they need to be confirmed by longitudinal studies. Additionally, studies are needed to test the relative and absolute harm and addictiveness of NPs to support or refute some of these beliefs. Furthermore, federal and state authorities need to continue surveying these beliefs and potentially develop necessary public health campaigns to correct potential misbeliefs about NPs.

Three correlates of NP use are notable. First, male adults were more likely than female adults to have ever used and currently be using NPs. Previous research suggests that while smokeless tobacco product use is more prevalent among men than women [16], both genders appear to use them for different reasons [45]. Therefore, researchers and public health officials should further investigate the reasons for using NPs among men and women. Second, young or middle-aged adults were more likely than older adults to report ever and current NP use. This is concerning, as the health impacts of these products on younger individuals, particularly among young adults whose brains are still developing, are unknown. Additionally, NP advertisements commonly promote these products as trendy through depictions of youthful models, appealing through an array of flavor options, easy and convenient as they do not require a device or inhalation, and healthy through messaging such as “tobacco-free” [24,42,46]. These messages may cause confusion and further increase the likelihood of use among this younger population, which is more vulnerable to nicotine addiction than older adults [44,47]. Third, current cigarette, electronic vaping products, and smokeless tobacco users were more likely than non-users to have ever used and currently be using NPs. If NP use is replacing the use of these other tobacco products, we would expect a higher prevalence of ever and current NPs use among *former* commercial tobacco users, not *current* commercial tobacco users. Our findings suggest that NPs may be co-used with other tobacco products rather than being used as a means of switching for harm reduction purposes or as a smoking/smokeless tobacco cessation aid. Future longitudinal studies are needed to illuminate our hypotheses.

The present study has limitations. First, our findings may not be generalizable to nicotine-naïve adults and youth. Future studies are needed to examine NP awareness, use, and beliefs among nicotine-naïve adults and youth to fully understand the public health impact of these novel tobacco products. Second, given the novelty of NPs, respondents may not fully understand what NPs are and found it difficult to answer the items about NP-related beliefs. We tried to mitigate this issue by providing respondents with pictures and descriptions of NPs and by including “don’t know” as an option for NP belief-related items so that respondents were not forced to agree or disagree with those items. Third, respondents were surveyed in 2021 during the COVID-19 pandemic. It is unclear how the pandemic may have affected respondents’ beliefs (particularly in relation to health risks) and use of NPs. Fourth, the data collected were from self-reports, and responses were anonymous. Self-report surveys and anonymous responses may present issues in terms of respondent bias or inaccurate reporting. Fifth, data were collected using YouGov online panel. While using an online panel does not capture individuals who do not have online access, YouGov is well respected and accepted in the field as a representative data source. Finally, due to the low prevalence of current NP use, some statistical comparisons may have insufficient statistical power to detect a small to medium effect size. As such, it is necessary to continue surveillance of NP use with large sample sizes to confirm our findings.

## 5. Conclusions

In conclusion, while NP use is currently low among US adults and current commercial tobacco users, close to half of this population were aware of these products, suggesting that NP awareness is on the rise. Additionally, a substantial proportion of this population holds positive beliefs about these products, and holding these positive beliefs is associated with susceptibility to and more advanced/higher levels of NP use. Given the NP companies’ effort in marketing their products to younger adults, restrictions on NP advertising could be necessary to prevent the co-use of NPs with other tobacco products, which could worsen nicotine addiction. Future studies are needed to examine the harm of NP use, and the role of NP use in combustible and smokeless tobacco use cessation. Continuing monitoring of NP awareness, use, and beliefs is warranted to determine the co-use of NP and other commercial tobacco products, as well as to understand NP’s roles in tobacco product use cessation. 

## Figures and Tables

**Table 1:** Weighted prevalence of nicotine pouches awareness, ever use, and current use and their associations with demographics and tobacco product use statuses, 2021 (N = 1583).

Variables		%	Aware of NP		Ever Used		Current Use	
			%	AOR (95% CI)	%	AOR (95% CI)	%	AOR (95% CI)
**Demographics**	**Age**							
	18–30 years	25.8	52.1	**2.93 (1.74, 4.96)**	20.8	**4.17 (1.78, 9.78)**	4.2	**54.72 (6.24, 480.21)**
	31–45 years	32.4	53.5	**2.99 (1.87, 4.80)**	26.0	**5.49 (2.47, 12.20)**	5.1	**58.76 (7.52, 459.23)**
	46–60 years	26.6	42.8	**1.87 (1.13, 3.08)**	6.2	0.98 (0.41, 2.37)	0.9	9.58 (0.88, 103.80)
	61+ years	15.2	29.4	Ref.	6.2	Ref.	0.1	Ref.
	**Sex**							
	Male	59.7	49.1	1.26 (0.92, 1.73)	18.6	1.47 (0.95, 2.29)	3.8	1.97 (0.88, 4.44)
	Female	40.3	42.9	Ref.	13.1	Ref.	1.7	Ref.
	**Race/Ethnicity**							
	Hispanic	13.2	44.4	0.74 (0.47, 1.16)	14.1	0.62 (0.36, 1.05)	3.0	0.92 (0.34, 2.49)
	Asian	3.7	43.0	0.67 (0.39, 1.18)	20.9	0.86 (0.42, 1.78)	5.7	1.24 (0.45, 3.37)
	Black	15.0	36.9	**0.60 (0.39, 0.93)**	13.4	0.72 (0.38, 1.36)	4.0	1.86 (0.52, 6.71)
	Other	2.6	43.8	0.75 (0.31, 1.81)	29.0	1.88 (0.64, 5.53)	0.0	Non-estimable
	White	65.4	49.6	Ref.	16.8	Ref.	2.7	Ref.
	**Educ.**							
	HS or less	48.4	45.6	1.05 (0.67, 1.65)	14.7	0.73 (0.39, 1.40)	1.8	0.59 (0.13, 2.73)
	Some college	34.0	48.7	1.14 (0.74, 1.73)	16.2	0.74 (0.43, 1.27)	3.6	1.02 (0.31, 3.34)
	College+	17.6	45.3	Ref.	21.3	Ref.	4.9	Ref.
	**Inc.**							
	<USD 50 K	61.1	45.7	0.91 (0.64, 1.30)	14.6	0.75 (0.44, 1.27)	1.8	0.41 (0.14, 1.22)
	%50 K+	38.9	48.4	Ref.	19.3	Ref.	4.8	Ref.
	**Urbancity**							
	Small city	9.3	44.8	1.14 (0.73, 1.78)	16.6	0.96 (0.55, 1.66)	2.4	0.59 (0.20, 1.78)
	Suburban	28.8	43.1	1.06 (0.70, 1.61)	15.8	0.81 (0.48, 1.36)	3.0	0.59 (0.23, 1.54)
	Small town	14.0	53.3	1.47 (0.87, 2.50)	18.1	0.99 (0.51, 1.95)	1.6	0.41 (0.13, 1.30)
	Rural	34.9	48.9	1.34 (0.83, 2.17)	15.0	0.87 (0.48, 1.59)	2.9	0.83 (0.24, 2.88)
	Big city	13.0	42.3	Ref.	19.1	Ref.	5.0	Ref.
**Tobacco products use status**	**Cigarettes**							
	Yes	74.9	44.7	0.84 (0.58, 1.22)	15.0	1.18 (0.72, 1.94)	3.1	**2.51 (1.02, 6.21)**
	No	25.1	52.1	Ref.	19.8	Ref.	2.7	Ref.
	**EVP**							
	Yes	30.4	51.8	1.05 (0.72, 1.54)	22.3	1.04 (0.64, 1.70)	6.6	**3.23 (1.07, 9.76)**
	No	69.6	44.3	Ref.	13.7	Ref.	1.4	Ref.
	**Cigars**							
	Yes	20.6	49.0	0.99 (0.64, 1.54)	23.3	0.80 (0.45, 1.42)	6.6	0.95 (0.29, 3.11)
	No	79.4	25.9	Ref.	14.5	Ref.	2.0	Ref.
	**Hookah**							
	Yes	11.0	45.2	0.61 (0.34, 1.10)	36.2	1.77 (0.92, 3.42)	9.6	1.11 (0.36, 3.40)
	No	89.0	46.7	Ref.	13.9	Ref.	2.1	Ref.
	**Other combus.**							
	Yes	15.7	29.5	1.57 (0.95, 2.58)	13.8	1.44 (0.77, 2.69)	7.9	0.78 (0.21, 3.05)
	No	84.3	44.9	Ref.	6.4	Ref.	2.0	Ref.
	**Smokeless**							
	Yes	13.8	56.8	1.31 (0.77, 2.23)	41.0	**3.36 (1.86, 6.09)**	12.8	**8.35 (2.64, 26.42)**
	No	86.2	45.0	Ref.	12.4	Ref.	1.4	Ref.

Boldface indicates statistical significance (*p* < 0.05). Inc. = income, Educ. = education, EVP = electronic vaping Product, NP = nicotine pouch, Other Combus. = other combustibles. Estimates for demographics are adjusted for demographics only. Estimates for tobacco products use status were adjusted for all variables in the table.

**Table 2:** Weighted prevalence of NP-related beliefs and their associations with nicotine pouch susceptibility and use status, 2021 (N = 1582).

Beliefs	Overall	NP Susceptibility and Use Status							
		Non-Susceptible Never (*n* = 627)		Susceptible Never (*n* = 677)		Ever-Not-Current (*n* = 215)		Current (*n* = 63)	
	% Agree	% Agree	AOR	% Agree	AOR (95% CI)	% Agree	AOR (95% CI)	% Agree	AOR (95% CI)
**NP is less harmful than smokeless tobacco**									
Agree	23.2	7.4	Ref.	32.7	**4.88 (2.44, 9.77)**	33.6	**2.91 (1.33, 6.34)**	76.3	**10.61 (3.44, 32.78)**
Don’t know	36.9	48.6	Ref.	33.7	1.12 (0.74, 1.69)	16.9	**0.40 (0.20, 0.79)**	0.4	**0.03 (0.00, 0.21)**
Disagree	39.9	44.0	Ref.	33.6	Ref.	49.4	Ref.	23.3	Ref.
**NP is less addictive than smokeless tobacco**									
Agree	16.1	4.0	Ref.	23.8	**4.11 (1.96, 8.63)**	20.8	**2.60 (1.12, 6.04)**	64.6	**12.74 (4.11, 39.47)**
Don’t know	38.1	49.9	Ref.	33.9	0.90 (0.61, 1.35)	21.2	**0.47 (0.25, 0.89)**	0.3	**0.01 (0.00, 0.12)**
Disagree	45.9	46.1	Ref.	42.3	Ref.	58.0	Ref.	35.1	Ref.
**Using NP occasionally is not harmful**									
Agree	18.9	5.6	Ref.	25.0	**3.49 (1.92, 6.37)**	30.4	**2.82 (1.37, 5.80)**	77.5	**14.24 (4.53, 44.78)**
Don’t know	37.1	48.4	Ref.	34.6	0.95 (0.63, 1.43)	16.3	**0.37 (0.19, 0.72)**	0.8	**0.05 (0.01, 0.30)**
Disagree	44.0	46.1	Ref.	40.4	Ref.	53.3	Ref.	21.7	Ref.
**Using NP occasionally is not addictive**									
Agree	14.6	3.8	Ref.	22.7	**3.70 (1.91, 7.19)**	15.7	1.27 (0.60, 2.68)	54.3	**6.63 (2.47, 17.76)**
Don’t know	36.5	49.3	Ref.	32.5	0.84 (0.57, 1.26)	15.3	**0.32 (0.16, 0.64)**	2.0	**0.09 (0.01, 0.71)**
Disagree	49.0	46.9	Ref.	44.8	Ref.	69.0	Ref.	43.8	Ref.
**NP is socially acceptable**									
Agree	33.2	15.2	Ref.	42.6	**3.93 (2.37, 6.50)**	50.2	**3.65 (1.97, 6.76)**	88.9	**23.07 (7.36, 72.28)**
Don’t know	34.7	48.3	Ref.	28.8	0.96 (0.62, 1.49)	16.1	0.49 (0.23, 1.03)	2.3	0.43 (0.06, 3.26)
Disagree	32.1	36.4	Ref.	28.6	Ref.	33.7	Ref.	8.8	Ref.
**NP is for someone like me**									
Agree	19.1	1.5	Ref.	25.5	**17.55 (7,78, 39.58)**	39.9	**24.78 (10.12, 60.68)**	93.7	**410.56 (54.89, >999.99)**
Don’t know	26.8	30.5	Ref.	29.4	**1.70 (1.11, 2.62)**	13.0	0.80 (0.38, 1.70)	0.7	0.46 (0.03, 6.53)
Disagree	54.1	68.0	Ref.	45.1	Ref.	47.1	Ref.	5.6	Ref.

Boldface indicates statistical significance (*p* < 0.05). NP = nicotine pouch. Each belief was modeled separately, adjusting for sociodemographics and tobacco products use statuses.

## Data Availability

Data can be made available upon request.
